# Intraspecific interference retards growth and development of cane toad tadpoles, but those effects disappear by the time of metamorphosis

**DOI:** 10.1098/rsos.231380

**Published:** 2023-11-08

**Authors:** M. R. Crossland, R. Shine

**Affiliations:** ^1^ School of Life and Environmental Sciences, University of Sydney, Sydney, New South Wales 2006, Australia; ^2^ School of Natural Sciences, Macquarie University, Sydney, New South Wales 2109, Australia

**Keywords:** body size, *Bufo marinus*, intraspecific competition, lifehistory

## Abstract

Competition among larval anurans can occur via interference as well as via a reduction in per-capita food supply. Previous research on intraspecific interference competition in cane toad (*Rhinella marina*) tadpoles found conflicting results, with one study detecting strong effects on tadpoles and another detecting no effects on metamorphs. A capacity to recover from competitive suppression by the time of metamorphosis might explain those contrasting impacts. In a laboratory experiment, we found that nine days of exposure to intraspecific interference competition strongly reduced tadpole growth and development, especially when the competing tadpoles were young (early-stage) individuals. Those competitive effects disappeared by the time of metamorphosis, with no significant effect of competition on metamorph body condition, size, larval period or survival. Temporal changes in the impact of competition were not related to tadpole density or to variation in water quality. The ability of larval cane toads to recover from intraspecific interference competition may enhance the invasive success of this species, because size at metamorphosis is a significant predictor of future fitness. Our study also demonstrates a cautionary tale: conclusions about the existence and strength of competitive interactions among anuran larvae may depend on which developmental stages are measured.

## 1. Introduction

Competition is a major factor structuring larval anuran communities and can occur via two broad types of mechanism: exploitation and/or interference [1,2]. Exploitation competition occurs via depletion of resources, such that the addition of conspecifics reduces per-capita food availability and thus, rates of growth, development and survival. Interference competition is less well-understood, because it may involve at least four mechanisms: (i) restriction of access to food owing to agonistic behaviour by other tadpoles, (ii) increased stress levels owing to interactions with other tadpoles, (iii) release of allelopathic chemicals or symbionts, and (iv) response to visual cues of competitors independent of physical or chemical interference [1,3–6]. Although anuran larvae have been model organisms for studying competition, few studies have attempted to separate the relative effects of exploitation versus interference mechanisms [2]. This is probably because interference effects can be difficult to evaluate, especially in the field [2]. Nonetheless, interference competition among anuran larvae has been documented in both laboratory and field experiments [1–4,7]. Additionally, interference competition can not only be an important determinant of fitness traits in anuran larvae, it may also be a key driver of selection (e.g. disruptive selection: [8]).

The cane toad (*Rhinella marina*), native to Central and tropical South America but introduced widely throughout the world as a biocontrol agent for insect pests of sugarcane crops [9], has been a popular study species for research on competition among tadpoles (Australia: [3,10–17]; Philippines: [18]; Japan: [19]; USA: [20]; Puerto Rico: [21]). Most of these studies have looked at overall impacts and thus have not attempted to separate the impacts of exploitation from those of interference, but two studies have explicitly assessed interference competition. Alford [3] demonstrated significant intraspecific interference competition (of unknown mechanisms) based on tadpole responses after 14 days' growth. By contrast, Cabrera-Guzmán *et al*. [14] found no evidence for chemically mediated intraspecific (or interspecific) interference competition based on impacts of larval competition on the traits of metamorph toads (age and size at metamorphosis). These contrasting findings could result from variation in methodology and/or tadpole clutches, or may indicate an ability of cane toad tadpoles to recover from intraspecific interference competition by the time they metamorphose. Although anuran larvae are known for their ability to recover from density-dependent effects of exploitation competition when competitor density is reduced (e.g. [17,22,23]), few data exist regarding their ability to recover from interference competition, particularly when there is no change in their exposure to interference cues during larval development. We conducted a laboratory experiment to assess the latter possibility. Additionally, we assessed whether the effects of intraspecific interference competition vary with the size/developmental stage of competing conspecific tadpoles.

## 2. Methods

We collected 12 adult cane toads (six male, six female) from the Adelaide River floodplain, Northern Territory and induced them to spawn by subcutaneous injection of synthetic gonadotrophin leuprorelin acetate (Lucrin, Abbot Australasia, 0.25 mg ml^−1^). Male toads were injected with 0.25 ml and female toads with 0.75 ml (as per [24–27]). Pairs of toads were placed in 80 l plastic tubs with a small amount of water and allowed to spawn overnight. We ultimately obtained two clutches from the six toad pairs injected in this manner, and randomly allocated each clutch as either the responding clutch (clutch A) or the stimulus clutch (clutch B; competitor clutch). For each clutch, eggs were transferred to separate 18 l plastic tubs filled with 9 l water, constantly aerated using small aquarium pumps with air stones attached. These tubs were located in an air-conditioned laboratory (25–26°C). As soon as embryos developed into free-swimming tadpoles, they were moved to outdoor 1000 l plastic bins filled with 750 l water and fed frozen lettuce and Algae Grazers pellets (HBH Products) ad libitum, with partial water changes every 2 to 3 days. Tadpoles from clutch A (approximately 400 tadpoles) were raised in one bin for 2 days, while tadpoles from clutch B were raised in two bins of differing densities (approx. 400 versus 1200 tadpoles) for 5 days to generate tadpoles of the same age but of different sizes and development stages. All tadpoles used in the experiment were haphazardly selected from these rearing bins. All water used in rearing bins and experimental tanks was groundwater sourced from a local aquifer. Tadpole development stages refer to the classification system of Gosner [28].

The experiment was conducted in a covered workshop exposed to ambient air temperatures at the Tropical Ecology Research Facility, Northern Territory (12.56° S, 131.32° E). Eighteen plastic tanks (65 cm × 41 cm × 39 cm) were divided in half using flyscreen mesh (1 mm × 1 mm), placed in 3 × 6 array (i.e. 3 treatments × 6 replicates), and filled with water to a depth of 33 cm. Ten tadpoles from clutch A were added to one randomly chosen side of each of tank, and exposed to one of three treatments: (i) 0 clutch B tadpoles (control), (ii) 10 small, early-stage clutch B tadpoles, or (iii) 10 large, later-stage clutch B tadpoles (see below for data on initial tadpole size and stage). Thus, for the experiment we used a total of 180 clutch A tadpoles, 60 small, early-stage clutch B tadpoles and 60 large, later-stage clutch B tadpoles. Allocation of treatments to tanks within blocks, and of tadpoles to tanks, was randomized. All clutch B tadpoles were placed on the opposite side of the mesh partition to clutch A tadpoles. This meant we could exclude exploitation competition as a mechanism for effects of clutch B on clutch A. Similarly, we could also exclude the possibility of interference competition via clutch B tadpoles physically restricting access to food by clutch A tadpoles, or by clutch B tadpoles increasing stress levels of clutch A tadpoles by close proximity or physical contact. Finally, we consider the possibility of visual interference to be unlikely because the only study to investigate this phenomenon found that visual cues of conspecifics (and heterospecifics) did not affect growth, development, or survival of *R. marina* tadpoles [14]. Thus, assuming no confounding effects of water quality among treatments (see Results), any competitive effects of clutch B tadpoles on clutch A tadpoles are probably owing to interference competition via release of allelopathic chemicals or symbionts.

At the start of the experiment, one tank within each treatment was randomly chosen and all 10 tadpoles were measured to obtain representative data on tadpole sizes/stages at the commencement of treatment exposure (clutch A tadpoles: 4.4–5.7 mm snout-vent length (SVL), 20–30 mg, stages 26–27; small early-stage clutch B tadpoles: 5.5–7.7 mm SVL, 30–60 mg, stages 28–30; large later stage clutch B tadpoles: 8.0–10.1 mm SVL, 80–150 mg, stages 31–36).

For the first 9 days of the experiment, tadpoles were fed blended Algae Grazers pellets every 3 days at a ratio of 3 mg tadpole^−1^ d^−1^; thereafter, this was increased to 4.5 mg tadpole^−1^ d^−1^ to account for increased food requirements of developing tadpoles. This feeding regime has been shown to be suitable for successful development of *R*. *marina* tadpoles [3]. On each feeding day, we counted the number of surviving tadpoles on both sides of each tank, and adjusted the amount of food added to each tank side accordingly. In this way, all tadpoles were exposed to a standardized amount of food. We measured water quality in each tank between 10.00 h and 14.00 h every 6 to 8 days: temperature and dissolved oxygen were measured using a YSI 85 meter, ammonia was measured using API Aquarium Pharmaceuticals test strips, and pH was measured using SSS Indicator pH paper.

We checked tanks daily and removed any dead tadpoles so they did not provide an additional food source. On day 9, we collected all clutch A tadpoles and recorded SVL (mm), mass (mg), developmental stage and survival. The tadpoles were then returned to their respective tanks and allowed to complete development. Metamorphosing clutch A tadpoles were collected and placed in 1 l plastic containers with a small amount of water. When metamorphosis was complete (defined as all four limbs emerged and tail fully resorbed), we measured snout-urostyle length (SUL; mm) and mass (mg), and calculated larval period from the start date of the experiment to the date when metamorphosis was complete. We also used the tadpole SVL and mass data, and metamorph SUL and mass data, to calculate the scaled mass index (SMI: [29]) for body condition for each tadpole and metamorph as follows:

$$\mathrm{scaled}\;\mathrm{mass}\;\mathrm{index}:\;{\hat{M}}_{i}=M_{i}{\left[\frac{L_{0}}{L_{i}}\right]}^{b_{\mathrm{SMA}}},$$

where *M*_i_ is individual body mass, *L*_i_ is individual body length (SVL for tadpoles, SUL for metamorphs), *L*_0_ is the average SVL or SUL of all individuals, and *b*_SMA_ is the scaling exponent. *b*_SMA_ is calculated by dividing the slope of the ordinary least squares regression of ln-transformed body mass on ln-transformed body length by the Pearson's correlation coefficient, *r*. For examples of the application of this body condition index to anurans see Zambrano-Fernández *et al*. [30] and Zamora-Camacho *et al*. [31].

We continued the experiment until all clutch A tadpoles had either died or metamorphosed, and recorded survival to metamorphosis for clutch A tadpoles in each tank. Clutch A tadpoles that died or metamorphosed during the experiment were not replaced. However, we did replace dead or metamorphosed clutch B tadpoles with siblings of similar size and stage to ensure that clutch A tadpoles were exposed to a constant density of clutch B tadpoles during their larval development. We did not change water during the experiment.

At the completion of the experiment, all surviving tadpoles and metamorphs were humanely euthanised using tricaine methanesulphonate (MS-222) as per our ethics approval. MS-222 is commonly used for euthanasia of amphibians (e.g. [24]).

### 2.1. Statistical analyses

We conducted all analyses in R [32]. Survival was scored as a binomial response (alive, dead) and analysed using logistic regression [33] and quasi-binomial models to account for overdispersion (mixed effects models: package MASS:glmmPQL, [34] followed by ANOVA package car, [35]). Other response variables (SMI, SVL, SUL, mass, development stage, larval period) were analysed using linear mixed-effects models (package nlme:lme, [36]). Linear mixed-effects models are useful for analysing ecological datasets because they are robust to violations in distribution assumptions that are a common feature of such datasets [37]. When overall treatment effects were significant, we conducted *post-hoc* multiple comparison Tukey tests adjusted with the Holm method (package multcomp, [38]). Specific details for analyses of data are given below.

### 2.2. Data on water quality

We did not formally analyse pH and ammonia data owing to obvious patterns in the data: pH was 6.0 in all tanks on all sampling dates, whereas ammonia was 0 mg l^−1^ in all tanks on day 8 and increased to 0.25 mg l^−1^ in all tanks over the remainder of the experiment. We analysed overall treatment effects on water temperature and dissolved oxygen using treatment and time as fixed effects, with tank nested within block as a random effect. Significant overall treatment effects were followed with multiple comparison tests for each sampling date using treatment as a fixed effect and block as a random effect.

### 2.3. Data on tadpoles and metamorphs

We analysed growth and development data for day 9 tadpoles (SMI, SVL, mass, development stage) and metamorphs (SMI, SUL, mass, larval period) using the fixed effect of treatment, with tank nested within block as a random effect. Larval period data were log-transformed prior to analysis to improve normality of residuals (package moments, [39]: raw data residuals skewness = 2.03 versus log-transformed data residuals skewness = 1.20). Survival data for day 9 tadpoles and metamorphs were analysed using the fixed effect of treatment, with block as a random effect.

## 3. Results

### 3.1. Water quality

**Figure 1.**
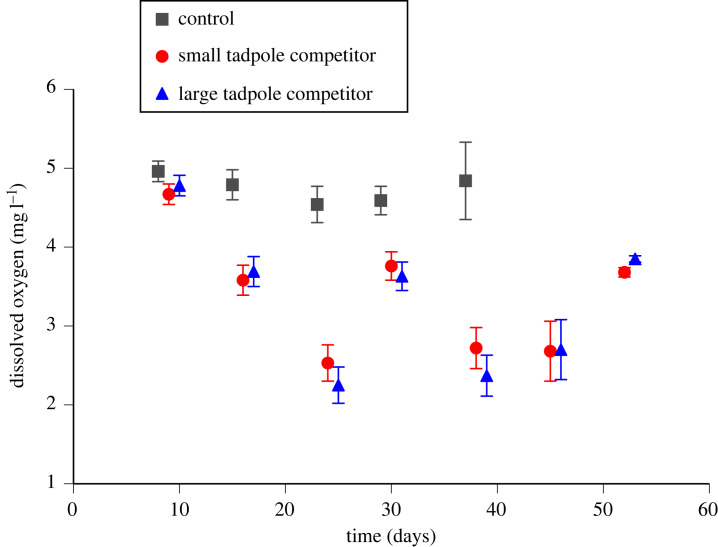
Levels of dissolved oxygen in treatment tanks throughout the experiment. Data plotted are mean ± standard error values. No data are presented for control tanks for days 44 and 51 because all control tadpoles had either metamorphosed or died before day 44.

Overall, water temperature did not vary significantly among treatments throughout the experiment, but dissolved oxygen did (table 1). At day 8 (the day before tadpoles were measured), dissolved oxygen did not vary significantly among treatments (*F*_2,10_ = 1.25 *p* = 0.33; figure 1) but from day 14 onwards, levels of dissolved oxygen were lower in the two competitor treatments than in the control (multiple comparisons: *p* < 0.003 in all cases) but the two competitor treatments did not differ from each other (multiple comparisons: *p* = 0.15 or greater in all cases; figure 1).

**Table 1.** Results for overall treatment effects on water temperature and dissolved oxygen throughout the experiment. (Significant *p*-values are highlighted in bold.)

	*F*	d.f.	*p*
total data			
water temperature			
treatment	0.16	2,10	0.855
time	0.05	1,67	0.831
dissolved oxygen			
treatment	26.613	2,10	**<0**.**0001**
time	23.594	1,67	**<0**.**0001**

### 3.2. Tadpoles at day 9

**Figure 2.**
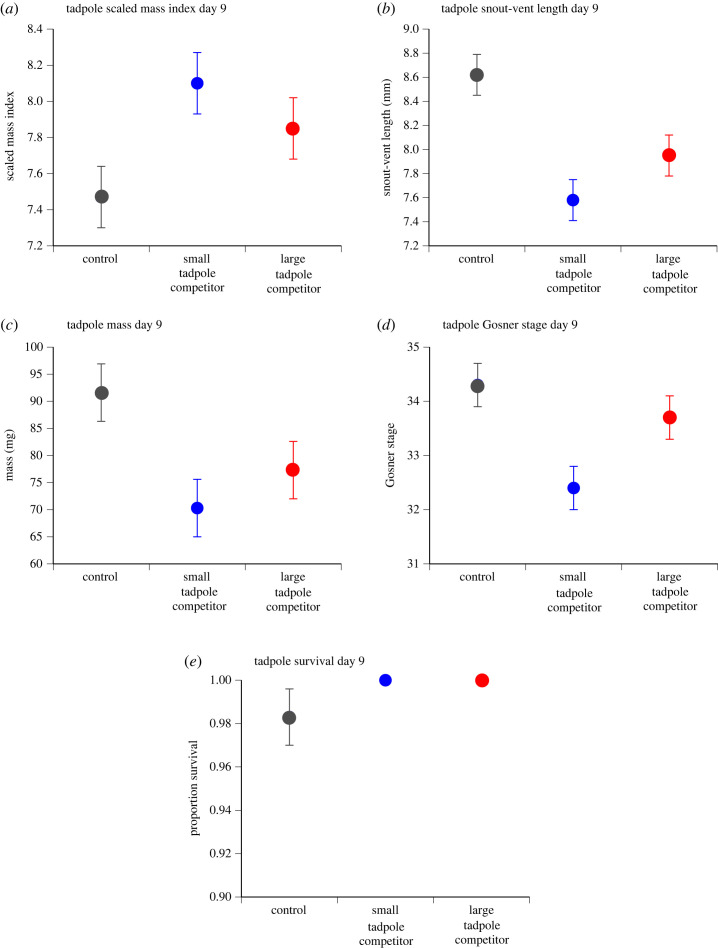
Treatment effects on day 9 tadpoles for (*a*) scaled mass index, (*b*) snout-vent length, (*c*) mass, (*d*) development stage [28] and (*e*) survival. Data plotted are mean ± standard error values.

Treatment affected mean tadpole SVL, mass and development stage at day 9, but did not affect SMI or survival (table 2 and figure 2). Multiple comparisons among treatments showed that responding tadpoles exposed to small (early-stage) competitor tadpoles had reduced SVL, mass and developmental stage relative to control tadpoles. By contrast, responding tadpoles exposed to large (later-stage) competitors had lower mean SVL than did control tadpoles, but mass and developmental stage were not significantly affected (table 3).

**Table 2.** Results for overall treatment effects on day 9 tadpoles. (Scaled mass index, snout-vent length, mass and development stage results are *F*-values. Survival result is chi-square value. Significant *p*-values are highlighted in bold.)

	*F*/chi-square	d.f.	*p*
scaled mass index	2.324	2,10	0.1483
snout-vent length	10.194	2,10	**0**.**0039**
mass	4.139	2,10	**0**.**049**
development stage	4.94	2,10	**0**.**0322**
survival	0	2	1

**Table 3.** Multiple comparison results for significant treatment effects on day 9 tadpoles. (Significant *p*-values are highlighted in bold.)

	*z*	*p*	effect size ± s.e.
snout-vent length			
small competitor versus control	−4.455	**<0**.**0001**	−1.05 ± 0.23
large competitor versus control	−2.874	**0**.**0081**	−0.67 ± 0.23
small competitor versus large competitor	−1.584	0.1132	−0.37 ± 0.23
mass			
small competitor versus control	−2.824	**0**.**0142**	−2.12 ± 0.75
large competitor versus control	−1.892	0.1169	−1.42 ± 0.75
small competitor versus large competitor	−0.932	0.3511	−0.70 ± 0.75
development stage			
small competitor versus control	−3.076	**0**.**0063**	−1.87 ± 0.61
large competitor versus control	−0.990	0.3220	−0.60 ± 0.61
small competitor versus large competitor	−2.092	0.0729	−1.27 ± 0.61

### 3.3. Metamorphs

**Figure 3.**
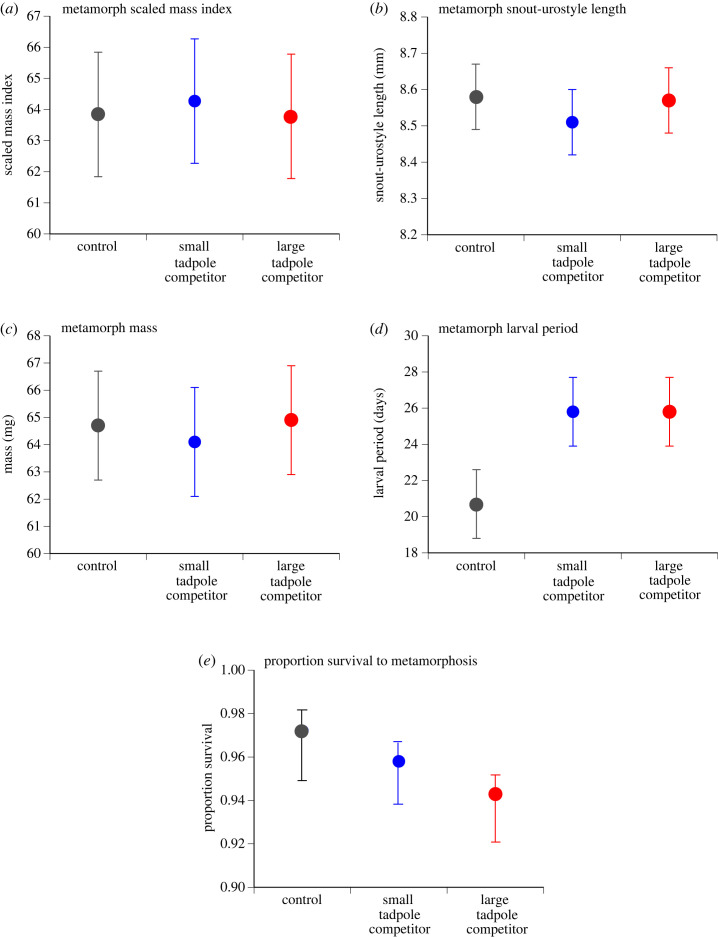
Treatment effects on metamorphs for (*a*) scaled mass index, (*b*) snout-urostyle length, (*c*) mass, (*d*) larval period (days to metamorphosis) and (*e*) survival. Data plotted are mean ± standard error values.

There were no significant effects of treatment on metamorph SMI, SUL, mass, larval period or survival (table 4 and figure 3).

**Table 4.** Results for overall treatment effects on metamorphs. (Scaled mass index, snout-urostyle length, mass and larval period results are *F*-values. Survival result is chi-square value.)

	*F*/chi-square	d.f.	*p*
scaled mass index	0.098	2,10	0.9078
snout-urostyle length	0.168	2,10	0.8479
mass	0.041	2,10	0.9602
larval period	2.725	2,10	0.1136
survival	0.833	2	0.6593

## 4. Discussion

Interpreting effects of competition among anuran larvae can be problematic because variation among experimental treatments can be confounded with variation in water quality [3]. Importantly, such effects were not a factor in our study. The only water quality parameter to show a treatment effect in our experiment (dissolved oxygen) is unlikely to have affected interpretation of our results. Dissolved oxygen levels were homogeneous on day 8, yet tadpoles that we measured the following day exhibited treatment effects on growth and development. Thereafter until the end of the experiment, dissolved oxygen levels were lower in both competitor treatments compared to the control but there were no significant treatment effects on growth, development or survival of metamorph toads. Because this reduction in dissolved oxygen was not associated with metamorph responses, treatment effects on water quality cannot explain our results.

Our study clarifies the contradictory results of Alford [3] (tadpole responses) and Cabrera-Guzmán *et al*. [14] (metamorph responses) for intraspecific interference competition among cane toad tadpoles in Australia: consistent with both these studies, we observed competitive effects during the tadpole stage, but these effects disappeared by the time of metamorphosis. Interestingly, although we detected significant treatment effects on tadpole SVL and mass at day 9, there was no significant treatment effect on body condition (SMI) at this time. Zambrano-Fernández *et al*. [30] found similar results for tadpoles of *Pelophylax perezi* where ammonium exposure significantly affected tadpole SVL and mass, but not SMI. This variation in responses suggests that treatment effects on morphology are proportional but do not affect the ability of tadpoles to store resources [30].

Given our study design (competing tadpoles separated by a mesh barrier, food allocated to tanks in a constant ratio relative to tadpole density), the interference competition effects we observed were probably via allelopathic chemicals or symbionts. Of these two possibilities, chemical interference seems more likely. Symbiont interference competition in anuran larvae is mediated by a microorganism excreted in the faeces of competitor tadpoles and consumed by the ‘target’ tadpoles [4]. For example, when competitor tadpoles are suspended in a mesh container above target tadpoles, the faeces of competitor tadpoles fall through the mesh and become available to the target tadpoles below for consumption (e.g. [4]). In our study the vertical mesh barrier between competing tadpoles would have made lateral movement of tadpole faeces between the two tank halves minimal to non-existent. Nonetheless, we cannot completely exclude this possibility (e.g. currents created by swimming tadpoles might move faeces between tank halves). Further work is required to determine the precise mechanism of interference competition documented in this study.

Body size can influence competitive interactions among anuran tadpoles, and when competition is via interference, larger individuals have a disproportionate advantage over smaller individuals when the former can physically interfere with the latter [23,40,41]. However, in our study where physical interference was prevented, competitive effects were stronger when the competing tadpoles were small (and early in development) rather than large (later in development). Few comparable data exist regarding the effect of size on non-physical interference competition in tadpoles. Where such data exist, they typically relate to the effect of body size on sensitivity to competitive effects. For example, Wong *et al*. [4] demonstrated that small, early-stage tadpoles of *Bufo calamita* and *Rana temporaria* were more prone to symbiont-mediated interference competitive effects than were larger, later-stage siblings. By contrast, our study on the effect of body size on non-physical interference revealed a negative relationship. Early-stage tadpoles may have more to gain by allocating effort to such intraspecific interference because they have more of their larval life remaining compared to larger (older) sibling tadpoles.

An ability to recover from early stress (including food deprivation) is widespread but not ubiquitous among animals. For example, in some lizards food availability early in life can have profound long-term impacts on an individual's subsequent growth trajectory and behaviour (e.g. [42] for *Zootoca vivipara*) whereas in other lizard species catch-up growth soon eradicates any short-term impacts of food supply in early life (e.g. [43] for *Amphibolurus muricatus*). For anuran larvae exposed to stressors during early life, significant growth recovery can occur once that stressor is removed (e.g. nutritional stress: [44,45]; salinity stress: [46]; temperature stress: [47]). However, other tadpole traits (e.g. development rate) may not recover to an equivalent extent [23,44]. Interestingly, growth responses may not be maintained across life stages. For example, *P. perezi* tadpoles experience increased mortality soon after hatching when exposed to ammonium. This increased mortality reduces one stressor (density) and despite ongoing exposure to the other stressor (ammonium), ammonium-exposed tadpoles are significantly larger than control conspecifics five weeks after hatching. However, this treatment effect disappears at metamorphosis, and is reversed in adult frogs [30,31]. The precise mechanisms that drive such effects remain to be determined but could involve compensatory growth [31]. Additionally, growth recovery can come with associated costs in other traits; for example, compensatory growth following a period of poor nutrition results in impaired cognitive performance in zebra finches (*Taeniopygia guttata*: [48]).

In anuran tadpoles, exploitative competition can reduce larval densities such that surviving larvae develop under more favourable conditions of food supply per capita, leading to growth recovery of the survivors and hence, a reduced impact of early food supply on traits at metamorphosis (e.g. [17,22,23]). Growth recovery (compensatory growth: a faster than usual growth rate, and/or catch-up growth: attainment of control size) following a period of dietary restriction has been demonstrated in a variety of taxa including arthropods, fishes, birds and mammals [49,50]. Our study indicates similar resilience of cane toad tadpoles to intraspecific interference competition, probably chemical interference. However, in contrast to density-mediated exploitation competition studies, we found that recovery from interference competition occurred despite the continued presence of the stressor (exposure to a constant density of competitor tadpoles throughout larval development). Reports of similar recovery from chemical interference seem to be rare and may be mediated by other factors. For example, female adult flour beetles (*Tribolium castaneum*) suppress each other's fecundity by chemical interference competition, specifically via toxic quinones secreted from their stink glands [51]. Such interference effects can be reversed without a change in density, but only if beetles are allowed to scavenge on nutrient-rich larval carcasses, which results in reduced quinone production inside the stink glands [51]. Throughout our experiment, competitor tadpoles (clutch B) were provided with a constant per-capita diet that did not vary in nutrient content. Thus, we can exclude the possibility of nutrient-driven changes in the potency of competitor tadpoles. Interestingly, this type of recovery from the negative impacts of intraspecific interactions is not seen for all stressors experienced during the early life stages of cane toads. Cane toad hatchlings accelerate development to escape cannibalism by older tadpoles, but this acceleration reduces subsequent growth, development, and survival. Tadpoles that initiate this accelerated development never recover even if raised in highly favourable conditions [26]. Given that body size at metamorphosis is a significant predictor of future growth and survival in cane toads [15], the capacity of cane toad tadpoles in Australia to recover from intraspecific interference competition may contribute to their successful colonization.

Our study was conducted with invasive cane toads in Australia. Indeed, all previous studies assessing competition involving cane toad tadpoles have been conducted in invasive populations (Australia: [3,10–17]; Philippines: [18]; Japan: [19]; USA: [20]; Puerto Rico: [21]). We are unaware of any published studies on competition involving cane toad tadpoles in their native range. Thus, we cannot assess whether the ability of cane toad tadpoles in Australia to recover from intraspecific interference competition is an ancestral trait or is a recent adaptation. However, given that cane toads have evolved numerous traits in the course of their Australian invasion (e.g. [52–54]), this possibility warrants further investigation, especially given records of geographical variation in the capacity for catch-up growth in other anurans (e.g. *R. temporaria* [47,55]). Additionally, we note that our experiment used tadpoles from two clutches, both of which were laid by adult toads collected in the Darwin rural area, Northern Territory. Relative to long-established populations of cane toads in eastern Australia (range-core), the toad population in the Darwin region can be considered to be just behind the ‘invasion front’. The growth and development of cane toad tadpoles from invasion-front populations in Australia is less sensitive to density effects compared to range-core populations [56]. Furthermore, cane toad tadpoles from invasion-front populations in Australia are less effective intraspecific competitors than are conspecifics from range-core populations [57]. Despite these competitive responses associated with invasion-front toads, the tadpoles in our study still showed significant capacity to recover from intraspecific interference competition. The extent to which our results are applicable to the broader invasion-front population remain to be determined. Similarly, differences in growth and development recovery between invasion-front versus range-core populations are yet to be assessed. However, given that range–core cane toad tadpoles are more sensitive to density effects and are stronger intraspecific competitors [56,57], it may be that range-core tadpoles have evolved even greater capacity for growth and development recovery from intraspecific interference competition compared to invasion-front toads.

In this study, we demonstrated the capacity of cane toad tadpoles to recover from competitive effects in the laboratory. To what extent can cane toad tadpoles recover from intraspecific interference (or exploitation) competition in the field? Unfortunately, no data exist to answer this question. Of the 13 studies that have investigated competition involving cane toad tadpoles (see previous paragraph), only one [15] was conducted in the field, and that study investigated interspecific competition (not intraspecific competition) and did not separate exploitation versus interference mechanisms or examine the capacity of toad tadpoles to recover from competitive effects. Indeed, few studies have attempted to quantify the relative contribution of interference versus exploitation competition among anuran larvae in general [2]. To add further complexity to the issue, additional factors such as water pH [58] and contaminants (pesticides: [18]; ammonium: [30]) may interact with competition among anuran larvae. Thus, further work is required to understand the relative importance of exploitation versus interference competition and the capacity for growth recovery for cane toad tadpoles in the wild.

Our results also offer a cautionary note: for any study aimed at quantifying competition among anuran larvae, results may depend upon the developmental stage at which data are recorded. In particular, studies based on short-term effects (during larval life) may overestimate subsequent impacts (at metamorphosis); and impacts measured at metamorphosis may fail to detect the ways in which competitive interactions can modify developmental trajectories during larval life.

## Data Availability

The data and R script are available on figshare: https://doi.org/10.6084/m9.figshare.24104010.v2 [59].
